# Functional Level and Dynamic Posturography Results Two Years after Vestibular Neurectomy in Patients with Severe Meniere’s Disease

**DOI:** 10.3390/jcm13123362

**Published:** 2024-06-07

**Authors:** Agnieszka Jasińska-Nowacka, Magdalena Lachowska, Kazimierz Niemczyk

**Affiliations:** Department of Otorhinolaryngology Head and Neck Surgery, Medical University of Warsaw, Banacha 1a Str., 02-097 Warsaw, Poland

**Keywords:** Meniere’s disease, vertigo, vestibular neurectomy, computerized dynamic posturography, vestibular compensation

## Abstract

**Objectives:** The aim of this study was to evaluate the functional outcomes and balance compensation in patients with severe Meniere’s disease after vestibular neurectomy. **Methods:** Pre- and postoperative results were analyzed in twenty patients with unilateral Meniere’s disease before and two years after vestibular neurectomy. Clinical evaluation was performed using a subjective grading scale proposed by the American Academy of Otolaryngology-Head and Neck Surgery and the Dizziness Handicap Inventory. Sensory organization test results were analyzed to assess the balance system before and after the surgery. **Results:** All patients reported a complete resolution of vertigo attacks after the vestibular neurectomy; 95% of patients reported functional level improvement according to a scale proposed by the American Academy of Otolaryngology-Head and Neck Surgery, and the average score decreased from 4.5 to 1.6. Clinical improvement, evaluated with the Dizziness Handicap Inventory, was present in all patients, with the average result decreasing from 81.7 to 16.4. Analyzing both grading systems, differences between pre- and postoperative results were statistically significant. No statistically significant differences were found between the sensory organization test results before and after vestibular neurectomy. Significant correlations were found between a patient’s age and postoperative results of the Dizziness Handicap Inventory and posturography. **Conclusions:** Vestibular neurectomy is an effective vertigo treatment in patients with severe Meniere’s disease with no clinical improvement despite conservative treatment. It results in subjective physical, functional, and emotional improvement, enabling patients to return to daily activities and work. An appropriate qualification of patients and comprehensive preoperative evaluation are essential to obtaining satisfactory clinical outcomes.

## 1. Introduction

Meniere’s disease (MD) is a chronic inner ear condition characterized by episodes of vertigo accompanied by sensorineural hearing loss, tinnitus, and aural fullness [1,2,3,4,5]. Attacks of spinning vertigo are caused by the pathological processes occurring in the inner ear affected by endolymphatic hydrops. The clinical course of MD is individually variable and unpredictable, with a frequency of spontaneous attacks ranging from less than once a year to daily [4,5,6,7]. International guidelines recommend an escalating approach in treating MD [8,9,10,11,12,13,14,15]. Initial therapy starts with lifestyle recommendations, including a low-salt diet, adequate water intake, avoiding alcohol, and caffeine intake reduction (<100 mg a day). The pharmacotherapy of MD begins with betahistine (first-line treatment in Europe) and diuretics. In patients with persistent vertigo and tinnitus, intratympanic injections may be applied as a second-line recommendation. Noninvasive treatment allows satisfactory clinical improvement in 80–90% of patients. Nevertheless, in patients complaining about disabling vertigo attacks despite conservative and intratympanic therapy, surgical treatment is indicated [3,8,9,10,11,12,13,14,15]. Among surgical techniques, we can distinguish procedures for preserving vestibular function (endolymphatic sac drainage and endolymphatic duct obliteration) and ablative surgeries (vestibular neurectomy and labyrinthectomy).

The main goal of treatment is to reduce vertigo episodes, considered the most impairing symptom of MD; thus, the therapy evaluation is based on the clinical outcomes, including changes in the frequency and severity of vertigo attacks [8]. Nevertheless, the chronic character of MD has an impact on patients’ daily activities, social life, and mental health during interattack periods as well. During the natural course of MD, exacerbations and remissions occur with individually variable patterns, resulting in anticipating anxiety and mood deterioration, including an increased risk of depression [16,17,18,19,20]; therefore, an assessment of a patient’s functional level and quality of life should be performed and repeated during the natural course of MD and after treatment.

The objective of the present study was to evaluate clinical outcomes and vestibular compensation in patients with clinically advanced MD treated with vestibular neurectomy. We aimed to analyze correlations between the subjective functional level and dynamic posturography results two years after surgery. An attempt has been made to identify predicting factors associated with poor vestibular compensation.

## 2. Materials and Methods

### 2.1. Ethical Consideration

The local University Ethics Committee reviewed and approved the study protocol (AKBE/65/2024 on 11 March 2024). Due to the retrospective study’s character, no written informed consent of participation was required. The project conforms to the Code of Ethics of the World Medical Association (Declaration of Helsinki).

### 2.2. Patient Description

Twenty patients with MD treated with vestibular neurectomy were enrolled in this retrospective study. All patients were diagnosed with unilateral definite MD according to the American Academy of Otolaryngology-Head and Neck Surgery (AAO-HNS) and Barany Society criteria [9]. To eliminate a differential diagnosis, such as vestibular schwannoma or central nervous system pathology, an MRI of the posterior fossa was performed. The patients demonstrated pharmacoresistant MD, defined as disabling vertigo attacks, despite a minimum of six months of conservative treatment, including lifestyle management, oral therapy with betahistine (48 mg a day), diuretic drugs, and intratympanic steroids (2 courses with four injections each). Patients presenting bilateral MD symptoms, a past medical history including chronic middle-ear conditions, a history of intratympanic gentamicin or prior ear surgery, and general anesthesia-related contradictions were excluded. A set of neurotological tests (clinical head impulse test, videonystagmography with caloric tests, and dynamic visual acuity) was performed before the surgery to assess the vestibular function precisely and to minimize the risk of postoperative bilateral vestibulopathy in cases of vestibular weakness present on the asymptomatic side. The decision of ablative surgical treatment was made individually for each patient due to intractable MD causing significant limitations in daily life activities caused by vertigo episodes (AAO-HNS functional scale ≥ 4) or Tumarkin’s attacks (also named an otolithic crisis, referring to drop attacks of vestibular origin that usually occur without warning).

### 2.3. Clinical Symptoms and Audiological Test Evaluation Protocol

A comprehensive clinical evaluation was performed in a tertiary care center in all analyzed patients before surgery. All the tests were performed during interattack periods. The same protocol was used during the follow-up visit 18–35 months after surgery.

Patients completed a retrospective questionnaire rating the intensity of their symptoms during the past six months. The average frequency of vertigo attacks per month was calculated. The intensity of tinnitus, aural fullness, and balance problems were self-assessed using a grading system ranging from 0 to 6, where the severity of symptoms increases with the scale proposed by Arenberg and Stahle [21]. Patients’ functional levels were self-evaluated using a 1 to 6 AAO-HNS scale (Table 1) [8]. Grades 3–6 were considered clinically advanced MD as patients must constantly adjust daily activities due to the symptoms [22]. Additionally, the impact of balance problems on daily life was assessed using the Dizziness Handicap Inventory (DHI)—Polish validated version [23,24]. The questionnaire includes 25 questions divided into three subcategories: physical (7 questions, 0–28 points), emotional (9 questions, 0–36 points), and functional (9 questions, 0–36 points). The total score can vary from 0 to 100 points. A DHI score of up to 14 points is considered normal, 16–34 points mild, 36–52 points moderate, and 54–100 points severe impairment (Table 2). After the surgery, a change of at least 18 points was considered clinically significant [25].

The pure tone average (PTA) hearing levels were calculated as the mean values among air conduction hearing threshold levels at 500, 1000, 2000, and 4000 Hz.

### 2.4. Balance Evaluation Protocol

Computerized dynamic posturography was performed before and after surgery using a NeuroCom device (EquiTest, NeuroCom; Clackamas, OR). The visual surround and the support may move, providing inaccurate information to the eyes, feet, and joints.

The sensory organization test (SOT) protocol included six tests measuring the sway of the center of gravity under six different conditions, each consisting of three 20 s trials:

Condition 1: eyes open, fixed support.

Condition 2: eyes closed, fixed support.

Condition 3: eyes open with sway-referenced visual surround, fixed support.

Condition 4: eyes open, sway-referenced support.

Condition 5: eyes closed, sway-referenced support.

Condition 6: eyes open with sway-referenced visual surround, sway-referenced support.

Conditions 5 and 6 are considered vestibular tests as the patient’s balance is exclusively based on the vestibular system due to the somatosensory and visual impairment in these conditions. The results of each condition can vary from 0 to 100. The composite equilibrium score, a weighted average of the six conditions, was calculated to evaluate the general balance level.

The sensory analysis ratios were calculated as follows to identify impairments of individual sensory systems:

(1) Somatosensory (SOM): the ratio of condition 2 to condition 1.
(2) Visual (VIS): the ratio of condition 4 to condition 1.
(3) Vestibular (VEST): the ratio of condition 5 to condition 1.
(4) Visual preference (PREF): ratio of conditions with unreliable vision (3 + 6) to conditions with absent vision (2 + 5).

Each sensory analysis ratio evaluates the ability to use specific senses in maintaining body posture, and the lower the parameter the more severe the impairment of the analyzed sensory system. The visual preference ratio defines if a patient can ignore inaccurate visual stimuli in a situation of sensory conflict. After analyzing each parameter, the system automatically establishes normative data for each test based on a patient’s age and height. In the present study, the SOT result was considered normal if the composite equilibrium score and all the sensory analysis results were normal.

### 2.5. Surgical Treatment

In all patients, a selective vestibular nerve section was performed by a very experienced otosurgeon (senior author of the manuscript—K.N.). The procedure was conducted through the middle fossa approach with the vestibular ganglion’s removal and preservation of cochlear and facial nerves. No dedicated vestibular rehabilitation was recommended beyond a gradual return to daily physical activity.

### 2.6. Statistical Analysis

Analysis of the relationships between patients’ clinical characteristics (age, disease duration, and the average number of vertigo attacks within the last six months before the surgery), pre- and postoperative results of subjective questionnaires (DHI total result, physical, functional, and emotional scores, and AAO-HNS functional level), and posturography scores (composite, VEST, and number of normal trials in the SOT) was performed. Changes between pre- and postoperative results were evaluated. A statistical analysis was conducted in the STATISTICA (data analysis software system), version 13 (TIBCO Software Inc. 2017, Santa Clara, CA, USA). The data were tested for normality, parametric, and non-parametric criteria. A detailed statistical analysis was performed using the Spearman’s rank and Wilcoxon signed-rank tests. The level of statistical significance was set at *p* = 0.05.

## 3. Results

### 3.1. Patients’ Characteristics

Twenty patients (thirteen females and seven males) with unilateral definite MD (13 in the left ear, 7 in the right ear) were enrolled in this study. Patients’ clinical data are presented in Table 3.

### 3.2. Preoperative Clinical Data

Within six months before surgery, patients reported 2.93 vertigo episodes per month on average, with results ranging from 0 to 11.66 attacks per month. Of the patients, 20% had a history of Tumarkin’s attacks.

**Figure 1:**
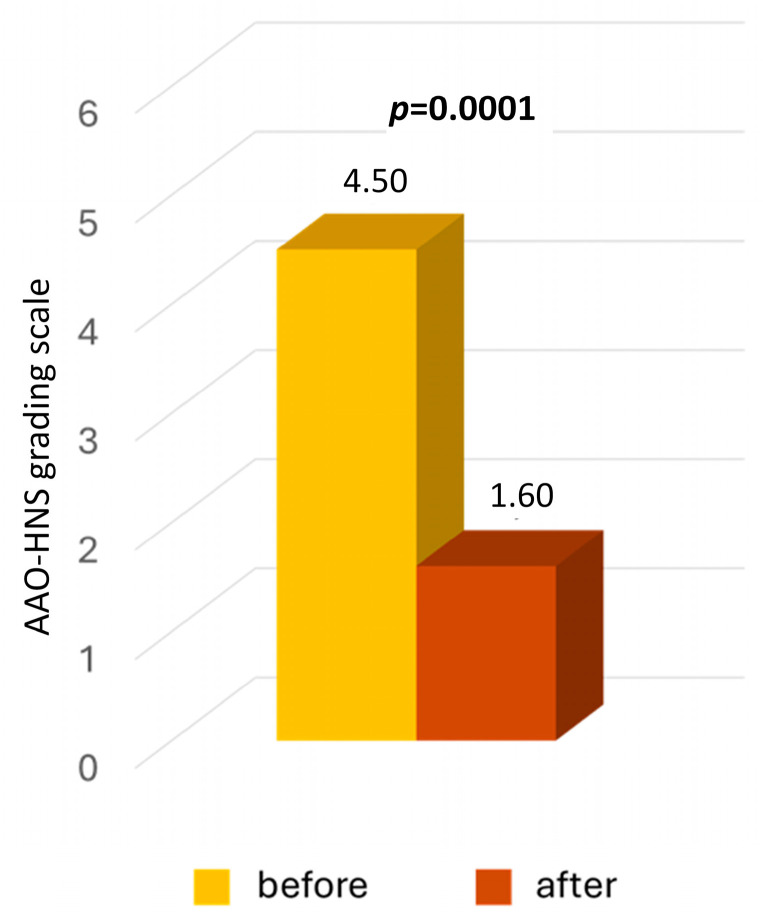
Functional level self-evaluated by patients with severe Meniere’s disease before and two years after middle fossa vestibular neurectomy. A grading scale ranging from 1 to 6, proposed by the American Academy of Otolaryngology-Head and Neck Surgery (AAO-HNS), was used. The *p*-value represents statistical significance.

**Figure 2:**
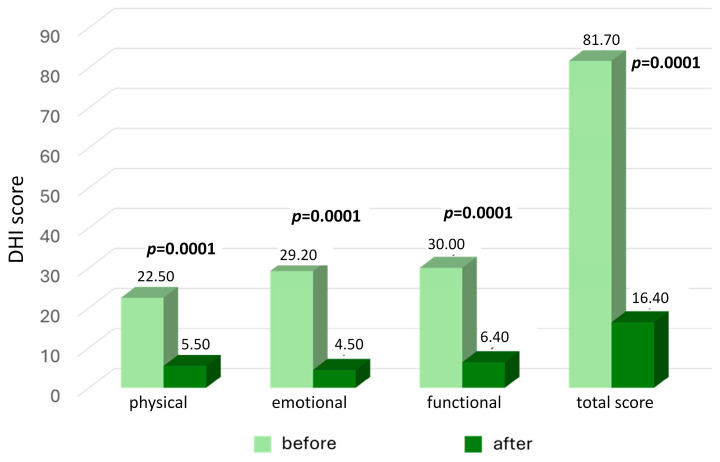
The results of the Dizziness Handicap Inventory (DHI) in patients with severe Meniere’s disease before and two years after middle fossa vestibular neurectomy. The *p*-value represents statistical significance. The *p*-value represents statistical significance.

The results of functional grading systems before and after surgery are presented in Table 3, as well as Figure 1 and Figure 2. Before surgery, using the 1–6 AAO-HNS classification, all patients self-assessed their functional level as grades 3 to 6 (corresponding to 15%, 25%, 55%, and 5% of patients, respectively). The average functional level before surgery was 4.5.

The average preoperative score of the DHI questionnaire was 81.70 points, with results ranging from 42 to 100. Impairment degree was classified as moderate in 5% of patients (one patient) and severe in 95%.

In pure-tone audiometry, sensorineural hearing loss was found in 95% of patients. Despite low-frequency hearing loss, normal PTA values were found in 5% of patients (one patient). The mean PTA level was 53 dB HL (±17.95 dB) in affected ears and 16.54 dB HL (±10.28 dB) in contralateral asymptomatic ears.

### 3.3. Preoperative Posturography Results

**Figure 3:**
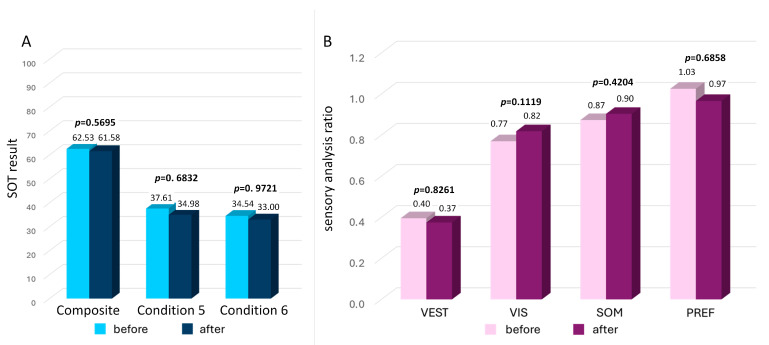
The results of the sensory organization test (SOT) in patients with severe Meniere’s disease before and two years after middle fossa vestibular neurectomy. Panel (**A**) general composite score (a weighted average of all the SOT results) and vestibular tests, condition 5 (eyes closed, sway-referenced support) and 6 (eyes open with sway-referenced visual surround, sway-referenced support) results. The *p*-value represents statistical significance. Panel (**B**) vestibular (VEST), visual (VIS), somatosensory (SOM), and visual preference (PREF) ratio results. The *p*-value represents statistical significance.

The results of computerized dynamic posturography before and after surgery are presented in Figure 3. Before surgery, normal SOT results were found in 30% of patients. In comparison, pathological results with at least one abnormal sensory analysis ratio or composite score were present in 65% of patients; 5% (one patient) did not complete the examination due to subjective discomfort unrelated to dizziness or vertigo. The average composite score was 62.53 (±17.65), with results ranging from 25 to 86. Normal and pathological composite balance scores were present in 35% and 60% of patients. The sensory analysis found decreased SOM, VIS, and VEST ratios in 25%, 40%, and 55% of patients, respectively. An abnormal PREF parameter was present in 15% of the study population.

### 3.4. Correlation between Clinical Data and Preoperative Results

An analysis of the relationships between patients’ clinical data (age, disease duration, and the average number of vertigo attacks within the last six months) and the preoperative diagnostic tests were made. A statistically significant correlation was found between the average number of vertigo episodes and the functional level evaluated using the AAO-HNS scale (*p* = 0.0068). No other relationships were found when analyzing subjective questionnaires (DHI total score, DHI physical, functional, and emotional scores). No statistically significant correlations were found between the patients’ clinical data mentioned above and SOT results (composite score, VEST ratio, and the total number of normal trials upon SOT examination).

### 3.5. Postoperative Clinical Data

All patients were discharged home in good general condition on the seventh day after surgery. The follow-up interval ranged from 18 to 35 months (mean: 25.11 months).

At the follow-up visit, all patients reported the complete resolution of vertigo attacks, with no episode for at least six months preceding the visit (class A vertigo control according to the AAO-HNS). No patient reported a Tumarkin’s crisis after the surgery. A reduction in balance problems was reported by 95% of patients, and the average intensity of balance problems decreased to 1.55 on the Arenberg scale. The mean tinnitus and aural fullness severity decreased to 3.48 and 1.12, respectively (Table 3). No patient reported aural symptoms in the contralateral ear.

According to the AAO-HNS 1–6 grading system, all patients self-assessed their postoperative functional level as grades 1 to 4 (corresponding to 55%, 35%, 5%, and 5% of patients, respectively), and 95% of patients reported an improved functional level. The average level was 1.6, and differences between pre- and postoperative results were statistically significant (*p* = 0.001) (Table 3, Figure 1).

After the surgery, the mean DHI total result was 16.4 points, with an average decrease of 65.3 points (Table 3, Figure 2). All patients reported a clinically significant improvement of at least 18 points. At the follow-up visit, normal DHI results were found in 50% of patients, and functional impairment degrees classified as mild and moderate were present in 45% and 5% of patients, respectively. None of the results were classified as a severe handicap. The mean results in the physical, functional, and emotional subcategories at the follow-up visits were 5.5, 6.4, and 4.5 points, respectively. Statistically significant differences between pre- and postoperative scores were found by analyzing the total DHI result, impairment degree, and each of the three subcategories (*p* = 0.001) (Figure 2).

The postoperative average PTA level in affected ears was 70 dB HL (±22.41 dB), with an average deterioration of 17 dB. The PTA level in contralateral asymptomatic ears was 17.38 dB HL (±11.60 dB).

### 3.6. Postoperative Posturography Results

At the follow-up visit, normal SOT results were present in 30% of patients. In comparison, in 65% of the study population at least one abnormal sensory analysis parameter or composite score was found. The normal result of the composite score was present in 30% of patients. The average composite score was 61.68 (±16.33), with results ranging from 26 to 87, and no significant difference was found compared to the preoperative examination (*p* = 0.5695). No statistically significant differences were found between the pre- and postoperative results of the vestibular tests (*p* = 0.6832 and *p* = 0.9721 for conditions 5 and 6, respectively) (Figure 3A). Additionally, the number of normal trials in each vestibular test was analyzed before and after the surgery, and no statistically significant differences were found (*p* = 0.8928 and *p* = 0.5940 for conditions 5 and 6, respectively). The sensory analysis found decreased SOM, VIS, and VEST results in 20%, 30%, and 55% of patients, respectively. After surgery, an abnormal PREF parameter was present in 20% of the study population. No statistically significant differences were found between the pre- and postoperative sensory analysis results, with *p* = 0.4204, *p* = 0.1119, *p* = 0.8261, and *p* = 0.6858 for SOM, VIS, VEST, and PREF ratios, respectively (Figure 3B).

### 3.7. Correlation between Clinical Data and Postoperative Results

Correlations were found between a patient’s age and postoperative total DHI results, as well as physical and functional DHI scores (*p* = 0.0106, *p* = 0.0194, and *p* = 0.0296, respectively), with no statistically significant relationship analyzing emotional DHI scores (*p* = 0.1077). A patient’s age did not correlate with the AAO-HNS functional level after surgery (*p* = 0.3588). After analyzing postoperative SOT results, significant negative relationships were found between the composite score and VEST parameters in correlation to a patient’s age (*p* = 0.0063 and *p* = 0.0345, respectively).

The disease duration did not correlate with postoperative functional level or SOT results after surgery.

The follow-up interval length did not correlate with postoperative functional level or SOT results after surgery.

Furthermore, an analysis of the relationships between a patient’s clinical data, mentioned above, and changes in functional level as well as SOT results before and after surgery was performed, and no statistically significant correlations were found.

Additionally, correlations were analyzed between the changes in self-assessed functional level evaluated in the DHI and AAO-HNS scales and SOT results; however, no statistically significant relationships were found.

## 4. Discussion

Vestibular neurectomy is known to be an effective treatment of disabling vertigo episodes in patients with severe MD with no clinical improvement despite conservative and intratympanic therapy. Several studies have reported vertigo control in 88–100% of patients [22,26,27,28,29,30,31,32,33,34]. In 1980, Garcia-Ibanez et al. [26] analyzed clinical outcomes in 373 patients three to seven years after middle fossa vestibular neurectomy. They reported complete relief of vertigo in 99.4% of patients with MD, while in the subpopulation of 41 patients suffering from other peripheral vestibular diseases the resolution of vertigo episodes was reported in 85.3%. Pappas et al. [22] analyzed the clinical results of vestibular neurectomy in a heterogeneous group of 41 patients suffering from MD, vestibular neuritis, and delayed endolymphatic hydrops. Two years after surgery, classes A and B of vertigo control were achieved in 70.73% and 14.63% of the study population, respectively.

Interestingly, satisfactory vertigo resolution (class A or B) was more often seen in patients with MD compared to others (90% vs. 80%), consistent with the abovementioned study. In a recent study by Veleine et al. [33], clinical outcomes were analyzed 10–16 years after vestibular neurectomy, and vertigo resolution was observed in 90.5% of patients. Leveque et al. [27] described clinical outcomes in 24 patients with MD after 6–50 months of follow-up and reported the complete resolution of vertigo episodes in 91.6% of patients. The authors analyzed clinical manifestations in two patients who reported persistent vertigo. They described benign paroxysmal positional vertigo (BPPV) of the posterior semicircular canal on the opposite side in one of them. At the same time, the second patient presented typical MD episodes on the operated side. In the present study, all patients reported total vertigo resolution at the two-year follow-up with no episodes within at least the last six months, classified as class A vertigo control according to the AAO-HNS [8]. Moreover, our results showed a significant correlation between the frequency of vertigo attacks and a patient’s functional level before surgery, which confirms that they are the most disabling MD manifestation that needs to be reduced.

Despite a satisfactory level of vertigo control, persistent balance problems are described in some patients after vestibular neurectomy [26,27,28,32,35]. Mild to moderate dizziness was reported by 33% of patients in the study by Leveque et al. [27], but only one patient considered it disabling. Similarly, chronic imbalance was observed in 33% of patients followed-up 2 to 15 years after neurectomy in the study by Thomsen et al. [32]. In the Garcia-Ibanez et al. [26] study, 9.4% of MD patients reported persistent balance problems three to seven years after middle fossa neurectomy. Similarly, Reid et al. [35] reported subjective chronic imbalance in 10% of patients with MD treated with vestibular neurectomy. They presumed that, in half of that subpopulation, the postoperative imbalance could be a manifestation of postoperative bilateral vestibulopathy due to asymptomatic contralateral vestibular weakness or MD development in the opposite ear. Thus, a comprehensive clinical evaluation is crucial before ablative therapy in patients with MD to avoid bilateral vestibulopathy. Presumably, a protocol including several vestibular tests evaluating the function of the semicircular canals (videonystagmography with caloric and rotational tests, head impulse test, and dynamic visual acuity), utricle and saccule (ocular and cervical vestibular evoked myogenic potentials), should be established. Nevertheless, the authors found no risk factors in the remaining half of patients demonstrating chronic postoperative imbalance.

Vestibular neurectomy involves the complete section of the afferent fibers, resulting in the symptomatic treatment of rotational vertigo in MD. Nevertheless, surgical labyrinth denervation induces acute postoperative asymmetry in the balance system, with unilateral static and dynamic vestibular deficit. Afterward, central nervous system reorganization should occur to enable vestibular compensation essential to physical and functional recovery [36]. The clinical course of the compensation is individually variable, and most studies describe that process using an animal model or acute unilateral vestibular loss in patients with vestibular neuritis [37,38]. Total surgical denervation of the labyrinth can be a model of vestibular compensation; however, a variable course of endolymphatic hydrops resulting in fluctuating vestibular function before the surgery makes interpreting postoperative compensation challenging. Further studies are necessary to better understand the vestibular compensation process in patients with MD.

Taking into consideration the fact that MD is associated with a higher risk of depression and social isolation, functional level and quality of life should be considered key aspects in evaluating the treatment’s effectiveness. Reid et al. [35] described clinical outcomes 1–10 years after vestibular nerve section in a heterogeneous group of 102 patients, including patients with MD and other peripheral-origin vertigo. The authors reported that 88% of patients were satisfied with clinical outcomes after the surgery. All patients analyzed by Garcia-Ibanez et al. [26] could work and perform normal activities despite postoperative balance problems. In the study by Thomsen et al. [32], among patients suffering from persistent balance problems after surgery, a preference for postoperative instability over the anxiety associated with the possibility of a vertigo attack was reported by all patients except for one. Interestingly, even the patient who underwent bilateral neurectomy preferred chronic symptoms resulting from bilateral vestibulopathy over episodic and unexpected rotatory Meniere’s attacks. Lemnos et al. [28] analyzed functional compensation after vestibular neurectomy in 15 patients with MD. Postoperative status was assessed one month and two years after surgery. At two-year follow-ups, 84.62% of patients reported the complete recovery of imbalance. In contrast, among the remaining group, one patient presented MD in the opposite ear, and another developed poor compensation, resulting in chronic imbalance. Of the patients, 46.15% reported working ability, 15.39% (two patients) did not work after the surgery, and information about the remaining patients is not included; however, comprehensive evaluations of patients’ functional levels were performed only at the last follow-up visit, which took place 24–95 months after surgery. To evaluate patients’ quality of life, authors used the Patients’ Global Impression of Change scale ranging from 1 to 7, where level 1 represents no change or subjective deterioration, and the level of improvement increases with the scale up to level 7, representing considerable improvement. At the last follow-up visit, 80% of patients reported a considerable improvement (level 7), and a noticeable change (level 5) was described by 13.33% of patients. In comparison, in 6.67% of the study population a change evaluated as level 2 was present (one patient with poor compensation). All 24 patients described by Leveque et al. [27] self-assessed that surgery improved their quality of life, enabling them to return to usual professional and social activities. In the study by Pappas et al. [22], two years after vestibular neurectomy, levels 1 and 2 in the AAO-HNS functional scale were reported in 70.73% and 9.76% of the study population, respectively. It is worth noting that, similar to the vertigo control analysis, satisfactory functional outcomes were seen more often in patients with MD than those with other balance disorders treated with vestibular neurectomy (84% vs. 70%). In the study by Vibert et al. [39], describing 19 patients suffering from clinically advanced MD with the preoperative AAO-HNS functional level ranging from 4 to 6, a satisfactory functional outcome with the postoperative result of level 2 maximum was achieved in 73.68% of patients two years after vestibular neurectomy.

In our study, 95% of patients reported improvement in the functional scale proposed by AAO-HNS, while in 5% (one patient) a stable functional status of grade 3 was present after surgery. Moreover, 90% of patients self-assessed their level as grade 1 or 2, indicating that patients could live without restrictions and do not have to change their daily plans because of MD symptoms. Since none of the patients had grade 5 or 6, we can assume that balance problems did not cause an inability to work after the surgery. Our results and the literature confirm that, after vestibular neurectomy, functional compensation is carried out, significantly improving a patient’s functional level.

Analyzing the DHI questionnaire, Lemnos et al. [28] reported normal or mild handicaps in 84.62% of patients at the last follow-up visit. Still, severe functional impairment was present in patients with MD in the contralateral ear and patients with poor compensation. Nevertheless, the authors did not include preoperative data regarding the DHI questionnaire, so the evaluation of functional compensation after surgery was limited. In our study, analyzing the DHI questionnaire, a clinically significant improvement of at least 18 points was reported by all patients two years after vestibular neurectomy. At the follow-up visit, normal DHI results were found in 50% of patients, and functional impairment degrees classified as mild and moderate were present in 45% and 5% of patients, respectively. None of the results was classified as a severe handicap. In contrast, before the surgery, 95% of patients reported severe impairment levels. Interestingly, correlations between a patient’s age and postoperative DHI total result in addition to the functional and physical scores were found, despite no relationships analyzing the preoperative questionnaires. Our results indicate that a patient’s age can impact the vestibular compensation course. This may result from deteriorating the efficiency of the central nervous system adaptation mechanisms. Our SOT results may support this hypothesis, as a patient’s age correlated significantly with the total balance composite score and VEST ratio after surgery. Interestingly, no relationship between a patient’s age and postoperative DHI emotional subcategory result indicates that processes caused by aging mainly impact the physical and functional aspects of vestibular compensation, while other factors may modify the emotional aspect. In our study, only patients up to the age of 65 were enrolled. Thus, a correlation between a patient’s age and postoperative results would presumably be observed as incomplete compensation in the elderly population. In these patients, some dedicated vestibular rehabilitation could be helpful to obtain optimal balance recovery after vestibular neurectomy. Further investigations on the elderly population are required to understand this subject better.

The usefulness of computerized dynamic posturography in diagnosing MD is limited, particularly due to the episodic character of symptoms. Soto et al. [40] reported a statistically significant variation in the interval since the last vertigo attack. Nevertheless, there is a strong relationship between balance problems and anxiety levels in patients with MD, which makes the balance system evaluation crucial in that population [41]. Moreover, it may be useful in the vestibular compensation assessment described in studies concerning patients after vestibular schwannoma surgeries [42,43]. In the present study, an analysis of computerized dynamic posturography found no significant differences between pre- and postoperative SOT results regarding the general composite balance score, vestibular test results, or VEST ratio. Presumably, a deterioration in SOT results would occur in the early postoperative period as a manifestation of acute vestibular loss. Importantly, preoperative diagnostics were performed in the interattack period so that we could evaluate the balance system outside vertigo episodes. At the two-year follow-up, no change in the composite score may be interpreted as compensation accomplished after the labyrinth denervation. After the surgery, no significant change in the VEST ratio was observed. The abnormal results of this parameter were still found in 55% of patients. This may suggest that patients’ ability to use the vestibular system to control their position and balance was significantly disturbed by endolymphatic hydrops before surgery, even during interattack periods. As the disease significantly disturbed the vestibular function, complete vestibular denervation resulted in vertigo relief without balance deterioration, which was observed in a long follow-up. Moreover, SOM, VIS, and PREF ratios were within the normal range in most patients, and no significant changes were found between pre- and postoperative results regarding these parameters. After vestibular denervation, the proper function of the somatosensory and visual senses is probably essential for balance compensation. Nevertheless, as the postoperative composite result was abnormal in 70% of patients, the objective balance deficit is presumably persistent and cannot be fully compensated for by the other senses.

The present study found no correlations between the subjective functional grading scales and posturography results. Similarly, Yip et al. [44] found no significant relationship between DHI score and posturography results in patients suffering from heterogeneous chronic vestibular diseases. The authors concluded that the DHI does not reflect the severity of the vestibular disorder. This may be particularly noticeable in patients with MD because of the episodic character of clinical symptoms resulting in fluctuating vestibular function. Consequently, during interattack periods, normal vestibular test results may not correlate with the functional level affected by persistent anxiety. Therefore, otoneurological examinations and functional level assessments are necessary for comprehensive clinical evaluation in patients with MD.

Our study found no correlations between the follow-up duration and the postoperative results, indicating that an 18-month period is sufficient for full vestibular compensation. In the other studies mentioned above, variable follow-up intervals were described; however, no correlation between postoperative results and precise follow-up durations was analyzed by the authors. Further studies with repeated visits could be helpful in better understanding the process of early compensation. Although we presume that vestibular compensation is accomplished within weeks to months after neurectomy, studies with longer follow-ups may help observe the development of MD in the contralateral ear [28,30].

In the present study, disease duration did not correlate with the functional level or SOT results before surgery, confirming MD’s individually variable clinical course with its nonlinear progression character [5,6,7]. Moreover, no relationships were found between the disease duration and postoperative results. Considering the satisfactory level of patients’ subjective functional compensation in the present study, we can assume that vestibular neurectomy can be applied successfully in patients with varying durations of MD, and decisions should be made individually.

Our study has potential limitations: First, the limited number of patients (total n = 20) is caused by the fact that less than 10% of patients with MD require surgical intervention and fulfill the inclusion criteria for this study. Moreover, the retrospective nature of the study negatively affects the level of evidence. In the present study, only one postoperative follow-up visit was analyzed, another limiting factor. Thus, further prospective research on a larger group of patients with several postoperative examinations is necessary to verify our results. Furthermore, other measures of functional outcomes and quality of life would help to better investigate clinical outcomes after vestibular neurectomy.

## 5. Conclusions

Vestibular neurectomy has excellent effectiveness in vertigo episode treatment in patients with severe MD. Moreover, it results in subjective physical, functional, and emotional improvement. Consequently, most patients self-assess their impairment level as significantly lower, enabling them to return to daily activities and work. The results of the present study indicate that functional vestibular compensation occurs spontaneously in most patients after vestibular denervation; however, we believe the appropriate qualification of patients and comprehensive preoperative evaluation are crucial to obtaining satisfactory clinical outcomes.

## Figures and Tables

**Table 1:** The functional level scale proposed by the American Academy of Otolaryngology-Head and Neck Surgery (AAO-HNS). Subjective questionnaire regarding the current state of overall function, not just functioning during attacks.

Grade	Patient’s Subjective Description
1	My dizziness has no effect on my activities at all
2	When I am dizzy I have to stop what I am doing for a while, but it soon passes and I can resume activities, I continue to work, drive, and engage in any activity I choose without restriction; I have not changed any plans or activities to accommodate my dizziness
3	When I am dizzy I have to stop what I am doing for a while, but it does pass and I can resume activities; I continue to work, drive, and engage in most activities I choose, but I have had to change some plans and make some allowance for my dizziness
4	I am able to work, drive, travel, take care of a family, or engage in most essential activities, but I must exert a great deal of effort to do so; I must constantly make adjustments in my activities and budget my energies; I am barely making it
5	I am unable to work, drive or take care of a family; I am unable to do most of the active things that I used to; even essential activities must be limited; I am disabled
6	I have been disabled for 1 year or longer and/or I receive compensation (money) because of my dizziness or balance

**Table 2:** Dizziness Handicap Inventory (DHI)—subjective questionnaire. Questions are divided into three subcategories: physical (P), functional (F), and emotional (E). The following scores can be assigned to each question depending on the patient’s answer: No = 0; sometimes = 2; and always = 4. Total score: 0–14 points = normal; 16–34 points = mild handicap; 36–52 points = moderate handicap; and 54–100 points = severe handicap.

P1. Does looking up increase your problem?
E2. Because of your problem, do you feel frustrated?
F3. Because of your problem, do you restrict your travel for business or recreation?
P4. Does walking down the aisle of a supermarket increase your problems?
F5. Because of your problem, do you have difficulty getting into or out of bed?
F6. Does your problem significantly restrict your participation in social activities, such as going out to dinner, going to the movies, dancing, or going to parties?
F7. Because of your problem, do you have difficulty reading?
P8. Does performing more ambitious activities such as sports, dancing, household chores (sweeping or putting dishes away) increase your problems?
E9. Because of your problem, are you afraid to leave your home without having someone accompany you?
E10. Because of your problem have you been embarrassed in front of others?
P11. Do quick movements of your head increase your problem?
F12. Because of your problem, do you avoid heights?
P13. Does turning over in bed increase your problem?
F14. Because of your problem, is it difficult for you to do strenuous homework or yard work?
E15. Because of your problem, are you afraid people may think you are intoxicated?
F16. Because of your problem, is it difficult for you to go for a walk by yourself?
P17. Does walking down a sidewalk increase your problem?
E18. Because of your problem, is it difficult for you to concentrate?
F19. Because of your problem, is it difficult for you to walk around your house in the dark?
E20. Because of your problem, are you afraid to stay home alone?
E21. Because of your problem, do you feel handicapped?
E22. Has the problem placed stress on your relationships with members of your family or friends?
E23. Because of your problem, are you depressed?
F24. Does your problem interfere with your job or household responsibilities?
P25. Does bending over increase your problem?

**Table 3:** Clinical characteristics and pre- and postoperative results of the analyzed patients with unilateral definite Ménière’s disease (MD) before and after vestibular neurectomy from the middle fossa approach. * Changes between pre- and postoperative results. AAO-HNS—American Academy of Otolaryngology-Head and Neck Surgery; DHI—Dizziness Handicap Inventory; SD—standard deviation; dB HL—decibels hearing level; min—minimum; and max—maximum.

**Before Surgery**					
**Patient’s Characteristics**		**Mean**	**Min**	**Max**	**SD**
Age (years)		50.60	34.00	65.00	11.60
MD duration (years)		6.93	1.00	18.00	4.68
Average number of vertigo episodes within the last 6 months (per month)		2.93	0.00	11.66	2.86
Balance problems	Average severity within the last 6 months (0–6 Arenberg scale)	3.07	0.00	5.50	1.27
Tinnitus		4.36	2.00	6.00	1.12
Aural fullness		3.02	0.00	6.00	1.71
Functional level (1–6 AAO-HNS scale)		4.50	3.00	6.00	0.83
DHI total score (0–100 points)		81.70	42.00	100.00	13.30
PTA level (dB HL)		53.00	17.50	78.75	17.95
**After Surgery**					
**Patient’s Characteristics**		**Mean**	**Min**	**Max**	**SD**
Follow-up interval (months)		25.11	18.00	35.00	3.88
Average number of vertigo episodes within the last 6 months (per month)		0.00 (−2.93) *	0.00	0.00	0.00
Balance problems	Average severity within the last 6 months (0–6 Arenberg scale)	1.55 (−1.52) *	0.00	4.00	1.33
Tinnitus		3.48 (−0.88) *	0.00	6.00	1.67
Aural fullness		1.12 (−1.90) *	0.00	5.50	1.47
Functional level (1–6 AAO-HNS scale)		1.60 (−2.90) *	1.00	4.00	0.82
DHI total score (0–100 points)		16.40 (−6.53) *	0.00	52.00	12.97
PTA level (dB HL)		70.00 (+17.00) *	41.25	120.00	22.41

## Data Availability

Dataset available on request from the authors.

## References

[1] Meniere P. (1861). Sur une forme de surdité grave dépendant d’une lésion de l’oreille interne. Gaz. Med. Paris.

[2] Gibson W.P.R. (2019). Meniere’s Disease. Adv. Otorhinolaryngol..

[3] Sajjadi H., Paparella M.M. (2008). Meniere’s disease. Lancet.

[4] Paparella M.M., Djalilian H.R. (2002). Etiology, pathophysiology of symptoms, and pathogenesis of Meniere’s disease. Otolaryngol. Clin. N. Am..

[5] Gurkov R., Pyyko I., Zou J., Kentala E. (2016). What is Meniere’s disease? A contemporary re-evaluation of endolymphatic hydrops. J. Neurol..

[6] Zhang Y., Liu B., Wang R., Jia R., Gu X. (2016). Characteristics of the Cochlear Symptoms and Functions in Meniere’s Disease. Chin. Med. J..

[7] Jasinska-Nowacka A., Lachowska M., Niemczyk K. (2023). Detailed clinical characteristics and its correlation with the diagnostic test results in patients with defined Ménière’s disease. Otolaryngol. Pol..

[8] Committee on Hearing and Equilibrium (1995). Committee on Hearing and Equilibrium guidelines for the diagnosis and evaluation of therapy in Meniere’s disease. Otolaryngol. Head Neck Surg..

[9] Lopez-Escamez J.A., Carey J., Chung W.-H., Goebel J.A., Magnusson M., Mandalà M., Newman-Toker D.E., Strupp M., Suzuki M., Trabalzini F. (2016). Diagnostic criteria for Meniere’s disease. Consensus document of the Barany Society, the Japan Society for Equilibrium Research, the European Academy of Otology and Neurotology (EAONO), the American Academy of Otolaryngology-Head and Neck Surgery (AAO-HNS) and the Korean Balance Society. Acta Otorrinolaringol. Esp..

[10] Basura G.J., Adams M.E., Monfared A., Schwartz S.R., Antonelli P.J., Burkard R., Bush M.L., Bykowski J., Colandrea M., Derebery J. (2020). Clinical Practice Guideline: Meniere’s Disease. Otolaryngol. Head Neck Surg..

[11] Nevoux J., Barbara M., Dornhoffer J., Gibson W., Kitahara T., Darrouzet V. (2018). International consensus (ICON) on treatment of Meniere’s disease. Eur. Ann. Otorhinolaryngol. Head Neck Dis..

[12] Nevoux J., Franco-Vidal V., Bouccara D., Parietti-Winkler C., Uziel A., Chays A., Dubernard X., Couloigner V., Darrouzet V., Mom T. (2017). Diagnostic and therapeutic strategy in Meniere’s disease. Guidelines of the French Otorhinolaryngology-Head and Neck Surgery Society (SFORL). Eur. Ann. Otorhinolaryngol. Head Neck Dis..

[13] Magnan J., Özgirgin O.N., Trabalzini F., Lacour M., Escamez A.L., Magnusson M., Güneri E.A., Guyot J.P., Nuti D., Mandalà M. (2018). European Position Statement on Diagnosis, and Treatment of Meniere’s Disease. J. Int. Adv. Otol..

[14] Quaranta N., Picciotti P., Porro G., Sterlicchio B., Danesi G., Petrone P., Asprella Libonati G. (2019). Therapeutic strategies in the treatment of Meniere’s disease: The Italian experience. Eur. Arch. Oto-Rhino-Laryngol..

[15] Niemczyk K., Jasińska A., Pierchała K. (2020). Ménière’s disease pt. 2. Treatment options and therapeutic strategies. Commentary to the current recommendations and own experience. Pol. Otorhinolaryngol. Rev..

[16] Lahiji M.R., Akbarpour M., Soleimani R., Asli R.H., Leyli E.K., Saberi A., Akbari M., Ramezani H., Nemati S. (2022). Prevalence of anxiety and depression in Meniere’s disease; a comparative analytical study. Am. J. Otolaryngol..

[17] Patel J.J., Levy D.A., Nguyen S.A., Rizk H.G., Meyer T.A. (2020). Depression in Meniere’s disease: A systematic review and meta-analysis. J. Laryngol. Otol..

[18] Bell S.L., Tyrrell J., Phoenix C. (2017). A day in the life of a Meniere’s patient: Understanding the lived experiences and mental health impacts of Meniere’s disease. Sociol. Health Illn..

[19] Tyrrell J., White M.P., Barrett G., Ronan N., Phoenix C., Whinney D.J., Osborne N.J. (2015). Mental Health and Subjective Well-being of Individuals With Meniere’s: Cross-sectional Analysis in the UK Biobank. Otol. Neurotol..

[20] Soderman A.C., Bergenius J., Bagger-Sjoback D., Tjell C., Langius A. (2001). Patients’ subjective evaluations of quality of life related to disease-specific symptoms, sense of coherence, and treatment in Meniere’s disease. Otol. Neurotol..

[21] Arenberg I.K., Stahle J. (1980). Staging Meniere’s disease (or any inner ear dysfunction) and the use of the vertigogram. Otolaryngol. Clin. N. Am..

[22] Pappas D.G., Pappas D.G. (1997). Vestibular nerve section: Long-term follow-up. Laryngoscope.

[23] Jacobson G.P., Newman C.W. (1990). The development of the Dizziness Handicap Inventory. Arch. Otolaryngol. Head Neck Surg..

[24] Szostek-Rogula S., Zamyslowska-Szmytke E. (2019). Validation of the Polish version of the Dizziness Handicap Inventory. Med. Pracy.

[25] Rizk H.G., Velozo C., Shah S., Hum M., Sharon J.D., McRackan T.R. (2024). Item Level Psychometrics of the Dizziness Handicap Inventory in Vestibular Migraine and Meniere’s Disease. Ear Hear..

[26] Garcia-Ibanez E., Garcia-Ibanez J.L. (1980). Middle fossa vestibular neurectomy: A report of 373 cases. Otolaryngol. Head Neck Surg..

[27] Leveque M., Seidermann L., Tran H., Langagne T., Ulmer E., Chays A. (2010). Vestibular function outcomes after vestibular neurectomy in Meniere disease: Can vestibular neurectomy provide complete vestibular deafferentation?. Auris Nasus Larynx.

[28] Lemnos L., Aubry K., Moreau J.J., Caire F., Salle H. (2019). Postoperative compensation after neurotomy in Meniere’s disease: Retrospective study of 15 cases. Neurochirurgie.

[29] Silverstein H., Wanamaker H., Flanzer J., Rosenberg S. (1992). Vestibular neurectomy in the United States—1990. Am. J. Otol..

[30] Schlegel M., Vibert D., Ott S.R., Hausler R., Caversaccio M.D. (2012). Functional results and quality of life after retrosigmoid vestibular neurectomy in patients with Meniere’s disease. Otol. Neurotol..

[31] Tewary A.K., Riley N., Kerr A.G. (1998). Long-term results of vestibular nerve section. J. Laryngol. Otol..

[32] Thomsen J., Berner B., Tos M. (2000). Vestibular neurectomy. Auris Nasus Larynx.

[33] Véleine Y., Brenet E., Labrousse M., Chays A., Bazin A., Kleiber J.C., Dubernard X. (2022). Long-term efficacy of vestibular neurotomy in disabling Meniere’s disease and Tumarkin drop attacks. J. Neurosurg..

[34] Jasinska-Nowacka A., Lachowska M., Wnuk E., Niemczyk K. (2022). Changes in endolymphatic hydrops after vestibular neurectomy observed in magnetic resonance imaging—A pilot study. Auris Nasus Larynx.

[35] Reid C.B., Eisenberg R., Halmagyi G.M., Fagan P.A. (1996). The outcome of vestibular nerve section for intractable vertigo: The patient’s point of view. Laryngoscope.

[36] Takeda N., Matsuda K., Fukuda J., Sato G., Uno A., Kitahara T. (2023). Vestibular compensation: Neural mechanisms and clinical implications for the treatment of vertigo. Auris Nasus Larynx..

[37] Esteban-Sanchez J., Martin-Sanz E. (2022). Long-Term Evolution of Vestibular Compensation, Postural Control, and Perceived Disability in a Population of Patients with Vestibular Neuritis. J. Clin. Med..

[38] Cass S.P., Goshgarian H.G. (1991). Vestibular compensation after labyrinthectomy and vestibular neurectomy in cats. Otolaryngol. Head Neck Surg..

[39] Vibert D., Allum J.H., Kompis M., Wiedmer S., Stieger C., Häusler R., Caversaccio M. (2018). Measurements of Trunk Sway for Stance and Gait Tasks 2 Years after Vestibular Neurectomy. Audiol. Neurootol..

[40] Soto A., Labella T., Santos S., Del Rio M., Lirola A., Cabanas E., Elhendi W. (2004). The usefulness of computerized dynamic posturography for the study of equilibrium in patients with Meniere’s disease: Correlation with clinical and audiologic data. Hear. Res..

[41] Miyazaki H., Nomura Y., Mardassi A., Deveze A., Miura M., Jike M., Magnan J. (2017). How minimally invasive vestibular neurotomy for incapacitating Meniere’s disease improves dizziness and anxiety. Acta Otolaryngol..

[42] Parietti-Winkler C., Gauchard G.C., Simon C., Perrin P.P. (2010). Long-term effects of vestibular compensation on balance control and sensory organisation after unilateral deafferentation due to vestibular schwannoma surgery. J. Neurol. Neurosurg. Psychiatry.

[43] Parietti-Winkler C., Gauchard G.C., Simon C., Perrin P.P. (2011). Pre-operative vestibular pattern and balance compensation after vestibular schwannoma surgery. Neuroscience.

[44] Yip C.W., Strupp M. (2018). The Dizziness Handicap Inventory does not correlate with vestibular function tests: A prospective study. J. Neurol..

